# Supplementary material for: Are carbon emissions trading and green financial instruments synergistic? -Comprehensive quantitative research based on content analysis
Source: PLoS One. 2024 Mar 7;19(3):e0298601. doi: 10.1371/journal.pone.0298601 (PMC10919601; doi:10.1371/journal.pone.0298601)
Supplement: S1 Data — (ZIP) [file pone.0298601.s001.zip › Data/地级市Z_F工作报告.zip!/║■▒▒/╛ú├┼╩╨/└┤╫╘╣1⁄2╓┌║┼í╛┬φ┐╦╩2╛▌═°í┐_2004.docx]

各位代表：

　　现在，我代表市人民政府向大会作工作报告，请予审议，并请各位政协委员和列席人员提出意见。

　　一、2003年工作回顾

　　过去的一年是不寻常的。在省委、省政府和市委的领导下，在市人大常委会、市政协的监督和支持下，市政府认真贯彻党的十六大和十六届三中全会精神，按照市人大六届一次会议确定的工作目标，紧紧依靠全市人民，顽强拼搏、团结奋进，战胜了汉江20年来最大一次洪水，克服了突如其来的“非典”疫情等不利影响，国民经济和社会发展取得新成绩。

　　(一)实施“兴工富市”战略成效显著。紧紧抓住发展第一要务，全面实施“兴工富市”战略，实现了经济较快增长。全市生产总值达到327.5亿元，增长9.4%。工业经济加快发展，整体效益明显改善。完成工业增加值130.8亿元，规模以上工业实现利税14.8亿元，分别增长11.9%和14.1%。化学、建材、纺织、食品、机电等传统产业改造步伐加快。生物医药、新材料、电子信息等高新技术产业快速发展。高新技术产业开发区和工业园区开发建设势头良好。骨干企业进一步发展壮大，全市销售收入逾亿元的工业企业达到28家，当年净增11家。坚持以工业理念发展农业产业化，农村经济结构调整取得明显进展。主要农产品除粮食因调减面积和受灾减产外，棉花、油料、肉类、水产品产量全面增长。农业结构不断优化，优质、无公害农产品生产规模继续扩大。农业产业化经营深入推进，一批龙头企业正在崛起。乡镇企业发展较快，完成增加值59.7亿元，增长30.7%。固定资产投资力度加大，发展后劲增强。全社会固定资产投资完成80.7亿元，增长15.1%。投资48亿元的荆门热电厂三期扩建工程和投资8亿元的京山永兴水泥项目，以及投资过亿元的金龙泉无纺布、新天科工业园、泰山建材、京山轻机工业园、京兰水泥、钟祥环锭纺二期、华溢纺织等项目，分别奠基、开工和投产。对外开放进一步扩大，招商引资取得丰硕成果。全年实际利用外资6285万美元，增长23.3%。外贸出口达到6686万美元，增长21.7%。财政金融运行平稳，经济活力进一步增强。地域财政收入20.6亿元，一般预算收入增长8.1 %。年末各类金融机构存贷款余额分别达到199亿元和139.1亿元，比年初增加28.9亿元和14亿元。全市实现社会消费品零售总额106.5亿元，增长10.2%。全市城镇居民人均可支配收入7059元，增长8.2%；农民人均纯收入3125元，增长4.2%。旅游、保险、房地产、现代物流、社区服务等服务业稳步发展。

　　(二)经济体制改革进一步深化。截至2003年底，市直绝大多数国有中小企业实行转制，金龙王等企业股权集中改革试点取得有益进展，非公有制经济得到新的发展。深化农村税费改革，减轻农民负担，同时实施乡镇综合配套改革，乡镇党政内设机构平均精简为6个，乡镇事业单位平均精减为7个。随着财税、金融体制改革全面展开，市县两级建立了较为完善的部门预算制度和国库集中收付制度。积极探索企业抵押担保和农户小额贷款方式，加大了对中小企业和农村经济的信贷支持力度。信用环境建设取得新成效，我市被授予全省“A级金融信用市”称号，钟祥市、京山县也被授予全省“最佳金融信用县市”称号。

　　(三)基础设施建设不断加强。围绕建设生态园林城市目标，坚持走经营城市之路，城市建设迈出新步伐，中心城区建设步伐加快，南城区开发建设势头良好。东宝山隧道全线贯通，三水厂管网、燃气管道工程和第二垃圾处理场建设稳步推进，天鹅广场小区和葡萄园城市花园等住宅小区建设进展顺利，城市综合功能不断增强。生态环境保护和建设力度加大，清理整顿和查处了违规排污企业，城市环境质量继续提高。我市被评为“中国优秀旅游城市”。交通、电力、通信等基础设施建设得到加强，高等级公路网建设全面展开，襄荆高速公路建设进展顺利，荆宜高速公路开工建设，武荆和经京山的随岳高速公路前期准备工作已经完成，207国道、汉宜路、安京路等改扩建工程全面竣工。信息化基础设施建设与应用取得新进展，广电宽带网二期建设全面启动，电信宽带网已基本覆盖荆门城区、县城和主要乡镇，全市互联网用户人数突破60万。

　　(四)社会各项事业全面发展。全社会科教、人才意识进一步增强，科技创新体系建设步伐加快，我市再次荣获“全国科技进步先进城市”称号。素质教育全面推进，高等教育、职业教育和成人教育得到加强，湖北理工学院筹建工作全面启动。社会科学、文学艺术、新闻出版、广播电视事业繁荣活跃。以建市20周年为契机，深入开展精神文明创建活动，极大地激发了全市人民的爱市热情。成功举办了“中国荆门郭店楚简”国际学术研讨会。社区文化活动丰富多彩，全民健身活动广泛开展。竞技体育水平不断提高，在省级以上运动会共获68枚金牌。公共卫生体系建设得到加强，成立了市、县两级疾病预防控制中心，建立了市紧急救援中心，提高了对重大疫病的防治能力。人口与计划生育工作不断强化，连续五年获全省综合评比一等奖。血防工作为全社会高度关注，48名晚血患者得到免费治疗。社会保障体系建设进一步加强，“两个确保”和“三条保障线”衔接工作进展顺利，全市养老、失业、医疗、工伤、生育等参保人数达76.4万人，发放社保资金3.54亿元。就业和再就业工作成效显著，全年新增就业岗位1.89万个，使1.6万名下岗失业人员实现了再就业。城市低保工作逐步规范，全市享受低保人员达9.4万人，发放低保资金5911万元，基本做到应保尽保。积极开展城乡救助，是民政对象和困难群体得到实惠最多的一年。加强社会治安综合治理，开展了以打击黑恶犯罪为重点的“严打”斗争，维护了社会稳定。国防动员、民兵预备役和人防工作取得新成效。国土、人事、审计、物价、统计、质量技术监督 、工商、民族宗教、对台事务、外事、侨务、气象、地方志、信访、保密、档案、老龄、残疾人等工作都取得了新进步。

　　(五)“服务型”政府建设迈出新步伐。认真学习邓小平理论、“三个代表”重要思想和党的十六大、十六届三中全会精神，不断改进政府工作，坚持向市人大及其常委会报告工作制度，重大决策征求市政协和民主党派、工商联、无党派人士以及人民团体的意见。全年办结、办复人大议案、代表建议和政协提案367件。加快转变政府职能，累计清理取消了522项行政审批事项，下放了18项市级经济管理权限，进一步放宽了民间资本市场准入领域。坚持依法行政、加强执法监督，政府工作人员的法制观念和依法办事能力有所增强。不断改进工作作风、转变行政服务方式，积极推行政务公开，开通“中国荆门”政府门户网站，开办“12345”市长专线，建立市行政服务中心、招投标交易中心、国库集中收付中心和经济发展环境投诉中心。着力创建“活跃的经济、廉洁的机关”，勤政廉政建设取得新成效。

　　各位代表，上述成就凝聚着全市人民的辛勤汗水。在此，请允许我代表市人民政府向全市各条战线的广大干部群众，向所有关心、支持和参与我市建设与发展的各界人士表示衷心的感谢并致以崇高的敬意！

　　在此，我们也清醒地认识到，在我市国民经济和社会生活中还存在一些突出的矛盾和问题，主要是：制约经济发展的体制性和结构性矛盾仍然比较突出，经济增长方式还没有得到根本转变，经济增长的质量和效益还不高；产业水平偏低、骨干企业偏少、发展后劲偏弱仍是制约城市综合竞争力的重要因素；农业产业化进展缓慢，农民收入增加不多，农业基础设施有待整固；社会保障制度建设与改革发展的要求不相适应，部分群众生活比较困难；社会不稳定的隐患依然存在，治安工作有待加强；少数公务员服务意识差，法制观念淡薄，不作为、乱作为现象仍有发生；经济环境有待进一步改善等等。对这些矛盾和问题，我们将努力采取有效措施，切实加以解决。

　　二、2004年主要工作

　　今年，是实现“十五”计划目标的关键之年，做好各项工作，意义十分重要。据此，今年政府工作的总体要求是：以邓小平理论和“三个代表”重要思想为指导，全面贯彻党的十六大、十六届三中全会精神，坚持以人为本、坚持科学的发展观、坚持以经济建设为中心、坚持“兴工富市”战略，重点做好结构调整、体制创新、项目建设和招商引资工作，切实转变政府职能、改进工作作风，正确处理改革、发展、稳定三者关系，最大限度地实现、维护和发展广大人民群众的根本利益，促进经济社会全面、协调、可持续发展。

　　根据上述要求，全市经济与社会发展的主要预期目标是：全市生产总值增长9.5%以上，规模以上工业增加值增长14%，全社会固定资产投资增长20%，社会消费品零售总额增长10%，外商投资增长20%，外贸出口增长15%，一般预算收入同口径增长10%，城镇居民人均可支配收入增长5%，农民人均纯收入增加150元以上，城镇新增就业岗位2万个，城镇登记失业率控制在4.5%以内，人口自然增长率控制在3‰以内。

　　为实现上述目标，将做好以下八项工作：

　　(一)大力实施“兴工富市”战略，坚持走新型工业化道路

　　认真组织实施《荆门市新型工业化规划纲要》，降低消耗、优化结构、改善品种、提高质量、增强竞争力，加快经济增长方式转变。坚持经济工作重点抓工业，工业重点抓投入，投入重点抓效益。切实做到领导力量向工业倾斜，主要精力向项目倾斜，工作重心向优势骨干企业倾斜，优厚待遇向经济人才倾斜，推动工业经济发展上新台阶。

　　延伸壮大产业链。按照“不论‘轻’‘重’、不求齐全、突出优势”的指导思想，加快工业经济结构调整，使重工业的优势进一步增强，轻工业的比重进一步提高，新兴产业蓬勃发展。坚持以信息化带动工业化，积极应用信息技术改造传统产业，延伸化工、食品、建材、机电等产业链。支持荆门石化、洋丰集团、百科药业、凯龙集团等企业的产品深度开发，推动传统化工向医药、精细化工转变；支持金龙泉集团等企业发展绿色保健食品，推动产品向系列化转变；支持葛洲坝股份有限公司水泥厂、光邦集团、荆玻集团等企业发展“四新两高”产品，推动传统建材向新型建材转变；支持京山轻机、东光集团等企业产品升级换代，推动光机电一体化。

　　做强做大骨干企业。继续实施“亿元工程”，以在荆中央、省属企业和地方骨干企业为龙头，充分发挥市场对资源配置的基础性作用，推动生产要素向支柱产业、优势行业、龙头企业、名牌产品集聚。重点在化肥、食品加工、新型建材等行业，培育一批主业突出、管理水平高、市场信誉好、竞争能力强的大公司、大集团，力争经过一段时间的努力，培植1家销售收入过100亿元的企业，形成销售收入过10亿、30亿、50亿元的企业梯队。坚持市县工业与乡镇企业共同发展，扶持一批技术含量高、管理能力强、市场前景好的中小企业迅速扩张。大力推动企业技术创新、管理创新和制度创新。鼓励和帮助企业积极应用信息技术改造生产工艺，推进生产过程自动化；改善企业管理，推进管理信息化；改进信息系统，推进知识管理网络化；改变营销手段，推进商务电子化。

　　加强工业园区和项目建设。集中精力抓好荆门高新技术产业开发区和京山、钟祥等地工业园区建设，加大项目引进力度。把工业园区逐步建成“兴工富市”的先进示范区、现代制造业的聚集区和新的经济增长极。加大重点项目建设力度，落实重点项目责任制。重点服务好荆门热电厂三期扩建工程、石化清洁燃料、金龙泉无纺布、宝源中高密度纤维板、钟祥环锭纺二期、京山永兴水泥、大峪口矿肥结合改造等项目建设。多渠道筹措工业发展资金，积极争取国债资金、高新技术产业化引导资金和其它国内外贷款。

　　(二)以增加农民收入为核心，全面发展农村经济

　　积极宣传、全面贯彻当前国家促进农业发展的各项政策，抓住机遇，有效地解决农业生产投入不足、基础设施老化、科技推广滞后和农民收入增加不多等突出问题，坚持“多予、少取、放活”和“扩大面积、主攻单产、加强植保、优化服务”的方针，以农民增收为核心，以结构调整为主线，进一步深化农村改革，落实各项政策，促进粮食增产、农业增效、农民增收。

　　加快农业结构调整。抓住国家实施优质粮食产业工程机遇，搞好钟祥、沙洋、京山三个国家优质专用粮食基地建设，稳定和提高粮食综合生产能力，确保全年粮食增产2.5亿公斤。坚持“市场引导、典型示范、自主种植”，推进板块经济建设，发展八大优势产业和八大特色产品。推行农业标准化生产，大力发展优质无公害食品、绿色食品和有机食品，不断提高农产品市场竞争力。

　　大力发展农村二、三产业。坚持“一主三化”方针，大力发展县域经济，以培育龙头企业为重点，大力发展农副产品深加工，延伸农业产业链，提高农副产品附加值，确保农民得到实惠。重点扶持宝源木业、金林木业、日月油脂、华联食品、国宝桥米、凤池米业和洪森实业等龙头企业，争取年内有1－2家企业进入国家级龙头企业行列。以农产品深加工和资源型企业为重点，加快发展乡镇企业，力争新增亿元乡镇企业5-8家。以发展农民专业合作组织为重点，大力发展农村第三产业。加强对农民工的培训和服务，支持和鼓励外出劳务。

　　充分调动和保护农民生产积极性。严格执行耕地保护政策。切实落实粮食“直补”、良种水稻补助和早中稻最低收购价保护政策，使农民得到实惠。做好取消农业特产税和降低农业税税率3个百分点的工作，锁定并逐步化解村级债务，切实减轻农民负担。市本级财政用于“三农”支出将达到1100万元，增长22%。还将积极争取和落实好国家对粮食生产、农业产业化、畜禽防疫、农民专业合作组织建设、农民培训、农业综合开发等专项资金。着力加强农村基础设施建设和农副产品流通市场建设，搞好支农信息技术服务。加强防汛抗灾，抓紧汉江堤防整治、病险水库除险加固、农田排灌体系和抗旱水源工程建设，抓好碾盘山水利枢纽工程和南水北调补偿工程建设，不断提高农业生产的基础保障能力。大力开展植树造林，继续搞好农村人口饮水和水土保持工程建设，切实改善农

　　业生态环境和农民生活环境。加强农产品检测检验和动物防疫体系建设，抓好高致病性禽流感等动物疫病防治工作。继续做好农村扶贫开发，特别是做好柴湖脱贫工作，稳步提高贫困地区农民生活水平。

　　(三)加快发展现代服务业，提高第三产业比重

　　积极发展旅游业。打好中国优秀旅游城市品牌，整合旅游资源，以“明显陵?大洪山”国债项目和荆门城区旅游圈为重点，实施旅游“畅通工程”。加大旅游项目招商引资力度，抓好重点景区开发建设，争取一批景区通过国家质量等级评定。推介一批精品旅游线路，实现旅游业发展新突破。大力发展现代物流业。深化流通企业改革，发展连锁经营、物流配送、电子商务等现代物流。加强专业市场、特色商业街区和农村连锁商业网点建设。加强市场监管，规范和维护市场秩序。加快发展信息服务业。继续推进“数字荆门”建设，积极发展电子政务和企业信息化，尽快形成覆盖全市的网络互联、信息交换平台，推动信息技术在国民经济和社会各领域更广泛的应用。大力发展房地产和社区服务业。加快经济适用房建设，培育二级市场，提高物业管理水平，推行住房分配货币

　　化，促进房地产业健康发展。完善社区服务设施，广泛开展社区服务，不断提高社区服务的质量和水平。

　　(四)深入推进各项改革，进一步扩大对外开放

　　着力推进产权制度改革。按照“归属清晰、权责明确、保护严格、流转顺畅”的要求，积极有效地盘活存量资产，推进现代产权制度建设。大力发展混合所有制经济。坚持“一视同仁、非禁即入”的原则，放宽民间资本市场准入领域，最大限度地开放投资主体，形成政府鼓励创业、群众自主创业的良好氛围。坚持投资项目市场化运作导向，充分发挥民间资本在基础设施和公用事业项目建设中的作用。大力促进、积极引导非公有制经济发展。

　　深化国有企业改革。认真妥善地处置企业改革过程中的各类矛盾和问题，进一步规范股份制改造，完善产权结构和法人治理结构、转换企业经营机制，通过资产重组与置换盘活存续企业。坚持依法规范的原则，制止借改革之名逃废债务和转移资产的违法行为，进一步落实国有资产经营责任，加强对上市公司监管，保证国有资产保值增值。按照“稳定区划、改革机构、精简人员、发展经济”的要求，推进国有农场深化改革。

　　加强社会保障体系建设。继续抓好“两个确保”和城市“低保”工作，在财政预算中增加社会保障支出，确保国有企业下岗职工基本生活费和离退休人员基本养老金按时发放，确保失业人员按规定足额享受失业保险金待遇。进一步扩大养老、失业、医疗、工伤、生育保险覆盖面，提高征缴率，力争新增扩面5万人。扩大农村贫困人口救助范围。高度重视就业再就业工作，认真落实再就业的各项政策措施，加大再就业资金投入，完善就业服务体系，强化再就业培训，建立再就业援助制度，扩大下岗失业人员再就业。

　　加快实施外向带动战略。坚持领导招商与专班招商相结合，形成招商引资的社会氛围。坚持以优势企业、优良资产、优质品牌和优良环境吸引外资。进一步改进招商引资方式，突出企业主体地位，充分发挥中介机构作用，探索招商引资市场化新途径。坚持市场多元化和以质取胜战略，努力扩大外贸出口。执行国家出口退税政策，优化出口结构，继续扶持东光、百科药业、京山轻机、山塔铁合金、永祥、普安等重点企业，增强出口竞争力。进一步扩大对外经济技术交流与合作，利用好“两个市场”、“两种资源”，拓展发展空间。

　　(五)认真做好财税金融工作，支持经济社会发展

　　加强公共财政体系建设。按照国务院的统一部署和省委、省政府的要求，继续推进、不断深化部门预算、国库集中收付制度、政府采购制度和“收支两条线”四项改革，构筑公共财政体制框架。严格依法治税，全面清理减免税，创新征管方式，规范税收秩序，做到应收尽收。积极培植新财源，巩固发展原有财源。牢固树立过紧日子的思想，按照公共财政的要求，“压一般支出，保工资兑现和机关运转；压机关消费支出，保群众基本生活；压养人支出，保公益事业发展”，优化支出结构，从严从紧安排和控制支出。高度重视社会各项事业发展，加大公益性事业投入，推进经营性事业机制创新。进一步加强审计监督，整顿财经纪律，规避、化解财政风险。

　　加大金融对经济发展的支持力度。进一步深化金融体制改革，优化信贷结构、改善金融服务。加大对骨干企业、中小企业和农业产业化龙头企业的信贷支持力度，做好下岗职工再就业的信贷扶持工作。完善社会信用体系，构建双赢互助的新型银企关系，以产业导向引导信贷流向。依照政策和法规，继续整顿、规范金融秩序，依法处理农村合作基金、供销社股金和非法集资等遗留问题，防范和化解金融风险。

　　(六)加强城市建设和管理，加速城乡一体化进程

　　抓好中心城区建设。坚持经营城市理念，按照“重点建设新区、适度改造旧城”的工作思路，加快东城区基础设施建设，完善城市功能。以路网、水网、气网、电网建设为重点，加大高新技术产业开发区和南城区开发力度。继续抓好三水厂管网配套、天然气管网工程和第二垃圾处理场建设，完善城市配套和服务设施。实施园林绿化工程，搞好东宝山公园、千佛洞森林公园和楚文化园建设，增加公共绿地，提升城市品位。放开城建投资市场，培育城市建设投融资主体，加快市政公用事业改革。

　　加强和规范城市管理。强化城市规划管理，根据经济社会发展趋势，抓紧做好荆门城市总体规划修编工作。支持各区根据城市总体规划积极参与城市建设，调动区政府管理城市的积极性和创造性。坚持“建管并举”，建立城市长效管理机制，不断提高城市规划、建设和管理水平。

　　加快城乡一体化进程。坚持城乡统筹发展，壮大县域经济。支持京山、钟祥两个县市扩权发展，支持沙洋、东宝、掇刀加快发展县（区）域经济。扶持一批基础条件好、发展潜力大的建制镇，把一批中心镇实力做强、规模做大、功能做全、形象做美，尽快形成具有竞争优势的经济强镇，充分发挥集聚、辐射和带动作用。大力实施“百村示范、千村整治”工程，逐步推进农村新社区建设，促进农民向市民转型、农村向社区转型，逐步形成城乡建设互动发展的新格局。

　　(七)积极发展社会各项事业，不断加强社会主义精神文明和民主法制建设

　　大力实施科教兴市和人才强市战略。积极推进科技进步和创新，认真组织实施一批高新技术产业项目，大力开发一批具有知识产权的技术和产品。加快高新技术特别是信息技术在新材料、食品、纺织等领域的应用。加强企业技术开发中心建设，继续推进产学研联合，促进科技成果的产业化、产业的规模化。健全农村科技服务体系，实施“乡有科技示范园、村有科技示范户、户有科技明白人”的“乡土人才”工程。坚持“科教兴市”，全面推进素质教育，提高教育质量和水平。巩固和完善以县级政府管理为主的农村义务教育管理体制，加大农村教育投入，改善办学条件、提高办学水平。加快湖北理工学院筹建步伐，做好申报和评估验收工作。发展多形式、多层次的职业技术教育和成人教育，完善继续教育制度，构建终身教育体系。鼓励和引导社会力量办学，规范和发展

　　民办教育。牢固树立人才资源是第一资源和以人为本的科学人才观，大力实施人才强市战略，坚持正确的用人导向，做到“不唯年龄看精神，不唯文凭看水平，不唯资历看业绩”，充分发挥各类人才在改革开放和现代化建设中的作用。进一步完善鼓励人才创新的制度和机制，重奖有突出贡献的各类人才，努力开创人才活力竞相迸发、聪明才智充分涌流的新局面。

　　加快发展社会各项事业。加快文化事业改革和发展，制定文化体制改革方案，完善文化产业政策，鼓励社会力量参与文化事业建设，努力形成先进的文化体制、发达的文化产业、一流的文化精品、繁荣的文化市场和丰富的文化生活。发展文学艺术、新闻出版、广播电视事业，实施全市广播电视“村村通”工程，做好文物保护工作。积极参加全国第十三届“群星奖”暨省第八届“楚天群星奖”竞赛活动，做好第八届中国艺术节荆门分会场筹备工作。进一步加强公共卫生体系建设，完善疾病预防控制体系、重大疫情信息网络体系和卫生执法监督体系，做好农村新型合作医疗试点工作，提高城乡医疗卫生保健水平，为人民群众提供多层次、多样化的卫生服务。广泛开展全民健身活动，提高竞技体育水平，积极备战省第十二届运动会。重视和做好民族宗教、信访、档案、保密、外事、侨务、对台事务、气象等工作。关心和支持妇女、儿童、老龄、残疾人等事业，做好关心下一代工作，促进社会和谐发展。

　　认真贯彻基本国策，坚持人口资源环境协调发展。加强人口和计划生育基础工作，高度重视并认真解决少数地方出生人口性别比失衡问题，改善人口结构、提高人口质量。科学开发和合理使用各类资源，健全资源有偿使用制度。加快退耕还林、天然林保护和长防林等重点生态工程建设。积极推行清洁生产，发展循环经济。按照全国统一部署，搞好第一次经济普查。

　　加强社会主义精神文明和民主法制建设。坚持以人为本，认真贯彻《公民道德建设实施纲要》，加强社会公德、职业道德和家庭美德教育，加强和改进未成年人思想道德建设，大力开展以“做文明公民、建诚信荆门”为主题的道德实践活动，广泛开展多种形式的文明创建活动，弘扬以爱国主义为核心的民族精神。大力表彰劳动模范，形成追求进步、学习先进的社会氛围。遵从人民群众期待公正、文明、科学、清明、富有创新活力的人文环境和具有鲜明时代精神的利益取向，依靠人民群众的积极性、主动性和创造性，推进先进生产力和先进文化的发展，不断提高劳动者的道德素质、科学素质和健康素质，实现人的全面发展。坚持“警民共建、携手联防、打防结合、预防为主”的方针，深入开展安全社区、单位、校园、乡镇和平安县市区创建活动。继续开展“严打”和“扫黄打非”斗争，坚决铲除黑恶势力和“黄赌毒”等社会丑恶现象，严厉打击“法轮功”等邪教组织。依法加强宗教管理，坚决取缔非法宗教组织和违法宗教活动。广泛开展国防教育，大力加强国防后备力量建设，深入推进“双拥”和民兵预备役工作，认真做好军队转业干部、复员退伍军人的接收安置和优抚工作。按照政策规定，积极做好老干部工作。高度重视人民群众来信来访和调解工作，努力把各种矛盾化解在萌芽状态。贯彻落实《劳动法》，加强劳动保护。高度重视安全生产，强化安全责任，防止重特大事故发生。

　　(八)切实改进工作作风，加强政府自身建设

　　加快转变政府职能。按照“精简、统一、效能”的原则，积极稳妥地推进市直机构调整，深化乡镇综合配套改革，加快推进事业单位改革，实现政企、政事分开。加快政府职能向“经济调节、社会管理、公共服务、市场监管”全面转换，努力建设服务型政府。深化行政审批制度改革，将该下放的权力一律下放、该取消的收费一律取消、该精简的审批一律精简。完善政府门户网站建设，完善“12345”市长专线和行政服务中心、招投标交易中心、国库集中收付中心和经济发展环境投诉中心的工作机制，为全社会提供优质、高效、便捷的服务。坚持政务信息公开制度，努力维护好群众知情权，增强政府工作透明度。加快信息化建设，大力发展电子政务，通过对计算机网络技术的应用，提高行政效率。

　　全面推进依法行政。认真学习宪法修正案，结合贯彻实施《中华人民共和国行政许可法》，坚持依法治市。深入开展普法教育，切实增强公务员的法律意识，提高依法施政能力。严格执法责任制和过错追究制，做到有权必有责、用权受监督、侵权要赔偿。规范法律服务、开展法律援助。加强执法队伍建设，提高执法人员素质，严格、公正、文明执法。

　　自觉接受人民监督。自觉接受同级人民代表大会及其常委会的监督，认真执行人大决议，接受人民政协的民主监督，认真听取民主党派、工商联、无党派人士和各人民团体的意见，推进民主科学决策。要尊重并接受新闻舆论和社会公众监督。支持人民群众通过行政复议、行政诉讼等法定渠道，监督政府机关及其工作人员。加强政府系统内部监督，支持监察、审计部门依法独立履行监督职责。

　　切实加强政风建设。全体公务员要牢固树立科学发展观和正确的政绩观，按照“为民、求实、清廉”的要求，大兴求真务实之风，鼓实劲、干实事、求实效，坚决纠正虚报浮夸、欺上瞒下、滥用民力、强迫命令等恶劣作风，坚决制止脱离实际的“政绩工程”、“形象工程”。下大力解决公共服务中的裙带关系、官僚主义、钱权交易、德能不称、部门不正之风等问题，使居其位而不谋其政、不尽其责者受到惩戒。认真解决在征用土地、城镇房屋拆迁、企业改制过程中政策不落实，以及拖欠和克扣民工工资等群众反映强烈的问题。坚持廉洁从政、从严治政，各级政府及政府组成人员必须牢记“两个务必”，认真贯彻落实两个《条例》，按照“四大纪律、八项要求”严格自律。全面落实廉政建设责任制，加大从源头预防和惩治腐败力度，建立不能腐败的防范机制，不敢腐败的惩治机制，不愿腐败的自律机制，以卓有成效的反腐倡廉成果取信于民。

　　各位代表，关心群众生活，努力为人民群众办实事，是各级政府应尽的职责。这里，我代表市人民政府郑重承诺，今年要着力办好以下十件实事，请各位代表和全市人民监督落实。一是确保城镇新增就业岗位2万个，帮助1500户双下岗失业人员家庭实现至少一人再就业。二是利用国债项目，建设县级公路242.5公里，新建通村四级公路450公里。三是改善市民就医条件，在城南新区新建一所综合医院。四是为全部危重血吸虫患者免费治疗，钉螺面积纯降1200亩，急感病人控制在4人次以内，加强对艾滋病的防治和对艾滋病人的帮扶。五是帮助2.4万名贫困中小学生就学，完成1.5万平方米农村中小学D级危房改造。六是改善市民生活环境，加强城区粉尘污染治理，城区环境空气质量达到国家二级标准以上的天数占85%以上，加强漳河水源环境保护工作，清理违章建筑，防治污染，保护植被。七是切实做好柴湖移民脱贫工作，确保实现年度工作目标。八是积极支持和帮助在荆中央、省属企业主辅分离、辅业改制工作，做好学校、医院等社会职能的移交工作，为中省企业发展创造良好的环境。九是继续解决农村人口饮水困难，并启动农村安全饮水工程，年内惠及1.5万人。十是定向援助2万农村特困人口，抓好农村五保“福星工程”，确保年内集中供养率达到80%以上。

　　各位代表！团结产生力量，廉洁凝聚人心，奋斗诞生兴旺！让我们紧密团结在以胡锦涛同志为总书记的党中央周围，在省委、省政府和市委的坚强领导下，高举邓小平理论和“三个代表”重要思想的伟大旗帜，与时俱进、奋发图强、开拓创新，加快实施“兴工富市”战略，为荆门经济社会全面、协调、可持续发展而努力奋斗！
